# Supplementary material for: An iPSC-derived astrocyte model of fragile X syndrome exhibits dysregulated cholesterol homeostasis
Source: Commun Biol. 2023 Jul 29;6:789. doi: 10.1038/s42003-023-05147-9 (PMC10387075; doi:10.1038/s42003-023-05147-9)
Supplement: Supplementary file 2 — Supplementary Information [file 42003_2023_5147_MOESM2_ESM.pdf]

Supplementary Figure 1.

An iPSC -derived astrocyte model of fragile X syndrome exhibits dysregulated cholesterol homeostasis

Talvio K, Wagner V, Minkeviciene R, Kirkwood JS, Kulinich AO, Umemori J, Bhatia A, Hur M, Käkelä, R, Ethell IM, and Castrén ML.

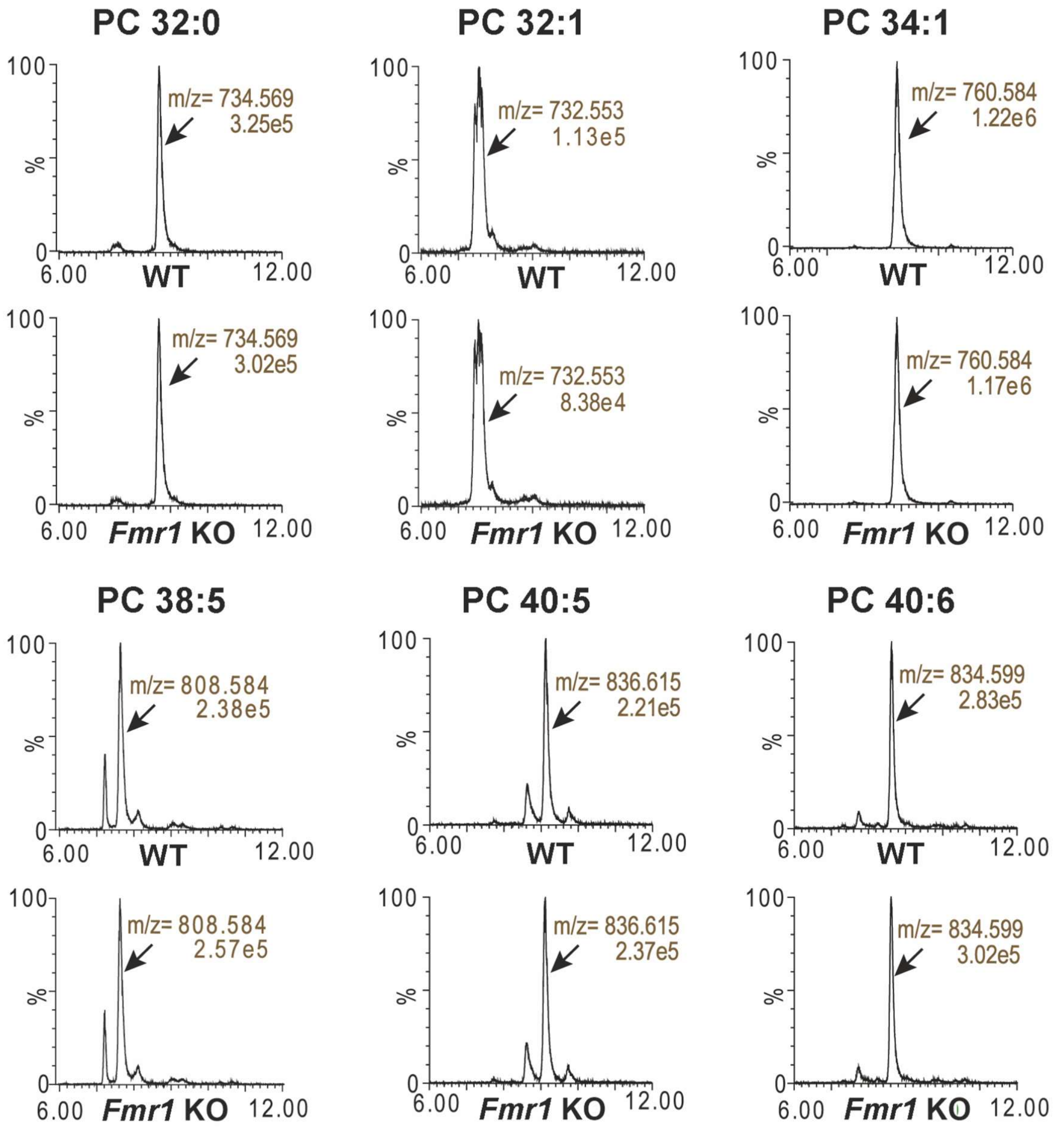

LC-MS ion chromatograms of representative *Fmr1* KO (FXS) and WT mouse astrocyte conditioned medium (ACM) samples with similar lipid concentration. The upper three paired figure panels indicate lower ion abundance (numbers below the m/z value) for the relatively short chain saturated and monounsaturated species of phosphatidylcholine (PC 32:0, PC 32:1 and PC 34:1) in the *Fmr1* KO ACM than in WT controls. The lower three paired figure panels indicate simultaneous higher ion abundance for the highly unsaturated species of phosphatidylcholine (PC 38:5, PC 40:5 and PC 40:6) in the *Fmr1* KO ACM sample. The corresponding quantitative PC species compositions are shown in Figure 2f.
